# Magnetic Resonance Imaging Features of the Sphenoid Sinus in Patients with Non-Functioning Pituitary Adenoma

**DOI:** 10.3390/medicina60050708

**Published:** 2024-04-25

**Authors:** Mircea-Viorel Ciurea, Ioan Ștefan Florian, Manuela Lenghel, Diana-Raluca Petea-Balea, Alexandra Roman, Silviu Albu

**Affiliations:** 1 Discipline of Oro-Maxillo-Facial Surgery and Implantology, Faculty of Dental Medicine, Iuliu Hatieganu University of Medicine and Pharmacy, 400029 Cluj-Napoca, Romania; 2 Department of Maxillo-Facial-Surgery, Cluj County Emergency Clinical Hospital, 400029 Cluj-Napoca, Romania; 3 Clinic of Neurosurgery, Cluj County Emergency Clinical Hospital, 400012 Cluj-Napoca, Romania; florian_stefan@umfcluj.com; 4 Department of Neurosurgery, Iuliu Hatieganu University of Medicine and Pharmacy, 400012 Cluj-Napoca, Romania; 5 Radiology Department, Iuliu Hatieganu University of Medicine and Pharmacy, 400012 Cluj-Napoca, Romania; pop.lavinia@umfcluj.ro (M.L.); diana.petea-balea@umfcluj.ro (D.-R.P.-B.); 6 Department of Periodontology, Faculty of Dental Medicine, Iuliu Hatieganu University of Medicine and Pharmacy, 400012 Cluj-Napoca, Romania; alexandra.roman@umfcluj.ro; 7 Emergency County Clinical Hospital Cluj, 400006 Cluj-Napoca, Romania; 8 2nd Department of Otolaryngology, Iuliu Hatieganu University of Medicine and Pharmacy, 400012 Cluj-Napoca, Romania; albu.silviu@umfcluj.ro

**Keywords:** magnetic resonance imaging, sphenoid sinus, non-functioning pituitary adenoma

## Abstract

*Background and Objectives*: A magnetic resonance imaging (MRI) scan is part of the diagnostic protocol in pituitary adenoma patients. The goal of the present study is to present and analyse the MRI appearances of the sphenoid sinus (SS) in patients with non-functioning pituitary adenoma (NFPA). *Materials and Methods*: This is a retrospective case–control study conducted between January 2015 and December 2023 in a tertiary referral hospital. Forty NFPA patients were included in the study group, while the control group consisted of 30 age- and gender-matched cases. *Results*: The sellar type of SS pneumatization was the most frequently encountered pattern among both groups. The presence of the lateral recess of the SS, mucosal cysts, and sphenoethmoidal cells was similar in both patient groups. The proportion of patients with SS mucosal thickness greater than 3 mm was 42.5% in NFPA group and 3% in the control group, and this difference was statistically significant (*p* < 0.001). The space between the two optic nerves was significantly larger in the NFPA group as compared to the control group (*p* < 0.001). *Conclusions*: Our study was able to establish a statistically significant association between the presence of NFPA and both the thickening of the SS mucosa and increased space between optic nerves.

## 1. Introduction

The endoscopic endonasal transsphenoidal approach (EEA) is the primary surgical technique for managing pituitary adenomas [1]. The sphenoid sinus (SS) holds significant importance as it serves as the primary passage to the pituitary fossa during transnasal transsphenoidal surgery. Positioned centrally within the skull base, the SS exhibits variability in size and shape. Despite advancements in intraoperative endoscopic and neuronavigation techniques enhancing the understanding of intrasphenoidal surgery and relevant cranial base procedures, they cannot replace the necessity for precise preoperative anatomical comprehension and assessment of neuroimaging studies [2]. Establishing a successful correlation between intraoperative anatomical observations and preoperative neuroimaging features is crucial for maximizing the safety of any endonasal approach to skull base surgery. The preoperative evaluation of sphenoid sinus anatomy via CT and magnetic resonance imaging (MRI) scans can aid surgeons in averting iatrogenic complications, such as damage to the internal carotid artery (ICA) and adjacent cranial nerves, during the sphenoid phase of surgery [3].

Non-functioning pituitary adenomas (NFPAs) are benign tumours originating from adenohypophyseal cells that are not associated with clinical or biochemical indications of hormonal excess [4]. The absence of symptoms related to hormonal overproduction typically results in a delay in diagnosis. Consequently, NFPAs are often identified when they reach a size significant enough to exert mass effects on nearby neurovascular structures [5].

In healthy persons, mucosal thickening is rarely encountered. In a recent study of healthy individuals aged 50 to 65 years, SS mucosal thickening was observed in only 7% of sinuses on MRI scans [6]. Discrepancies in the incidence of sphenoid sinus (SS) mucosal thickening on preoperative MRI scans among patients diagnosed with pituitary apoplexy (PA) and Rathke’s cleft cyst have been reported [7,8,9,10,11]. In a recent paper, Şahin B et al. assessed the relationship between NFPAs and SS parameters (mucosal thickening, cysts, lateral recess, distances between carotid arteries and optic nerves) on MRI scans [5]. The objective of this study was to evaluate the MRI characteristics of the SS and associated anatomic structures in patients with NFPA and compare them to those of a control group.

## 2. Materials and Methods

This study was conducted in accordance with the guidelines of the Declaration of Helsinki. The measurement protocols for this study were approved by the Iuliu Hatieganu University of Medicine and Pharmacy Cluj-Napoca Ethics Committee under approval No. 156/05 July 2023.

### 2.1. Patients

This is a retrospective case–control study conducted between January 2015 and December 2023. The study group included 40 patients operated on for non-functioning pituitary adenomas (NFPAs) in the Neurosurgery Department, a tertiary referral hospital. The control group consisted in 30 age- and gender-matched patients selected from the outpatient clinic of the IInd Otolaryngology Department. 

The diagnosis of NFPA was based on contrast-enhanced MRI of the pituitary gland and pituitary hormone levels within normal levels. The endocrinological evaluation of patients from the present study involved assessment of blood levels of growth hormone (GH), prolactin, free T4 (fT4), thyroid-stimulating hormone (TSH), insulin-like growth factor-1 (IGF-1), adrenocorticotropic hormone, cortisol, luteinising hormone, follicle-stimulating hormone (FSH), oestradiol, and progesterone (females) or testosterone (females and males).

The control group was selected from patients referred to the outpatient clinic for persistent headache, vertigo, asymmetrical hearing loss, or tinnitus and whose pituitary MRI scans and hormone levels were within normal parameters. Excluded from the study were patients with prior diagnosis of NFPA, chronic rhinosinusitis (diagnosis based on history, nasal endoscopy, and CT scan), previous endonasal surgery, nasal tumours, primary hypothyroidism, primary prolactin secreting pituitary tumour, previous head trauma, patients with a history of skull base or pituitary surgery and patients employing dopamine agonists, somatostatin analogues, oral contraceptives, testosterone, or oestrogens.

### 2.2. Magnetic Resonance Imaging Study

MRI of the brain and paranasal sinuses was performed on 1.5 Tesla Siemens scanner (Siemens AG, Erlangen, Germany) using an 8-channel head coil for all the patients included in the study. The study protocol included axial, coronal, and sagittal thin-sections on T1-weighted, T1 post-contrast, and T2-weighted sequences. Gadolinium-based contrast agent was employed during the study. The measurements were performed by a senior radiologist blinded to the clinical report. Different parameters of the SS were carefully documented: pneumatisation pattern (conchal type, presellar type, and sellar type), the presence of retention cysts, lateral recess, and the existence of the sphenoethmoidal air cell (Onodi). Mucosal thickening of the SS was measured on coronal T2-weighted sequences. According to a previous report [7], mucosal thickening above 1 mm was regarded as abnormal, and was split into three categories: ≤1 mm, between 1–3 mm, and ≥3 mm. As previously described in the study of Sahin et al., the intercarotid distance was determined at two separate points—petrous and cavernous [5]. The cavernous intercarotid length was estimated at the midportion of the cavernous section, while the petrous intercarotid distance was assessed at the joining of the horizontal and vertical segments of the petrous division. The space between the two optic nerves was assessed at the most typical cross-section of each nerve right in front of the optic chiasm on T2-weighed coronal sections. Additionally, on T2-weighted coronal images, the space between the two foramen rotundum was also evaluated. Furthermore, on T2-weighted coronal images, the adenoid tissue thickness was calculated from the superior wall of the nasopharynx.

### 2.3. Statistical Analysis

Data were displayed as means ± standard deviations (SD). The normal distribution of the analysed data was determined by the Kolmogorov–Smirnov (K-S) test for normality. For normally distributed data, the independent sample *t*-test was used to compare differences. Categorical variables were compared with chi-square test and Fisher exact test. Records were analysed using the SPSS statistical software, version 25.0.0 (SPSS Inc., Chicago, IL) and we considered the value of *p* < 0.05 as statistically significant. In order to observe a significant difference of 30% between controls and study group, concerning patients with SS mucosal thickening, a sample size of 30 patients was calculated using the two-sided Wilcoxon two-sample test at 5% level of significance, to give the study a statistical power of 80%.

## 3. Results

The average age in the study group was 42.5 ± 14.7 years while that in the control group was 44.7 ± 15.4 years. The difference is not statistically significant (*p* = 0.214). There were 25 females in the NFPA group and 17 females in the control group. No significant differences were noted in the gender distribution between the two groups (*p* = 0.622).

Abnormal mucosal thickening of the sinuses in both study and control group is depicted in Table 1. One can perceive that there are no significant differences in the proportion of abnormal sinus mucosa between the two groups, except for the sphenoid sinuses (SS). In contrast to the rest of paranasal sinuses, a statistical difference is demonstrated between the number of SS with thickened mucosa in the two groups. Mucosal thickness of ≥3 mm of the SS was observed in a greater proportion of the NFPA patients (42.5%) than in the control group (3%), and the difference is statistically significant.

The association between abnormal mucosal thickening of the sinuses and gender and season is depicted in Table 2. We confirmed a significant increase in the number of abnormal maxillary and anterior ethmoid sinuses in male patients as compared with females. However, there is no statistically significant association between gender and sphenoid sinuses mucosal thickening. There is no statistical difference between winter and summer in connection with abnormal sinuses mucosa on MRI.

**Figure 1:**
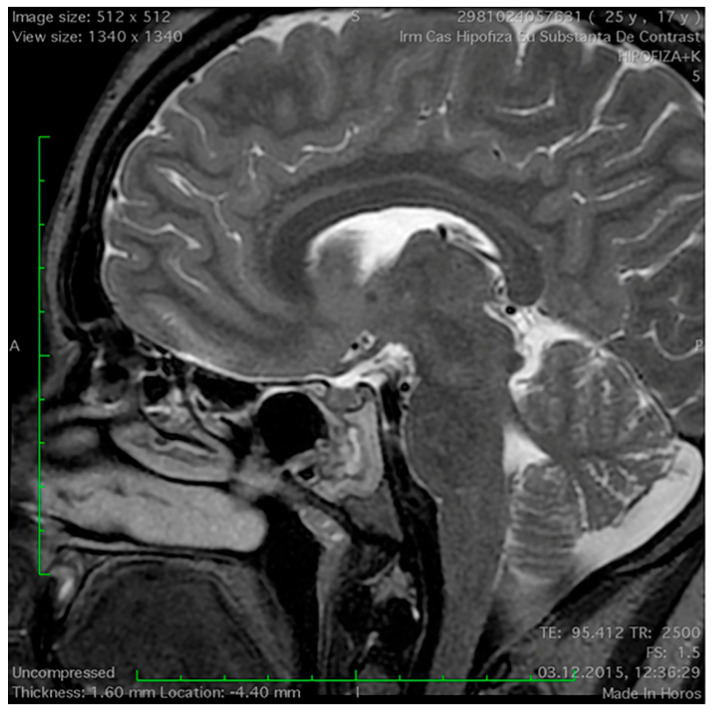
Mucosal thickening of the sphenoid sinus on a sagittal T2−weighted magnetic resonance imaging section in a patient with pituitary adenoma.

**Figure 2:**
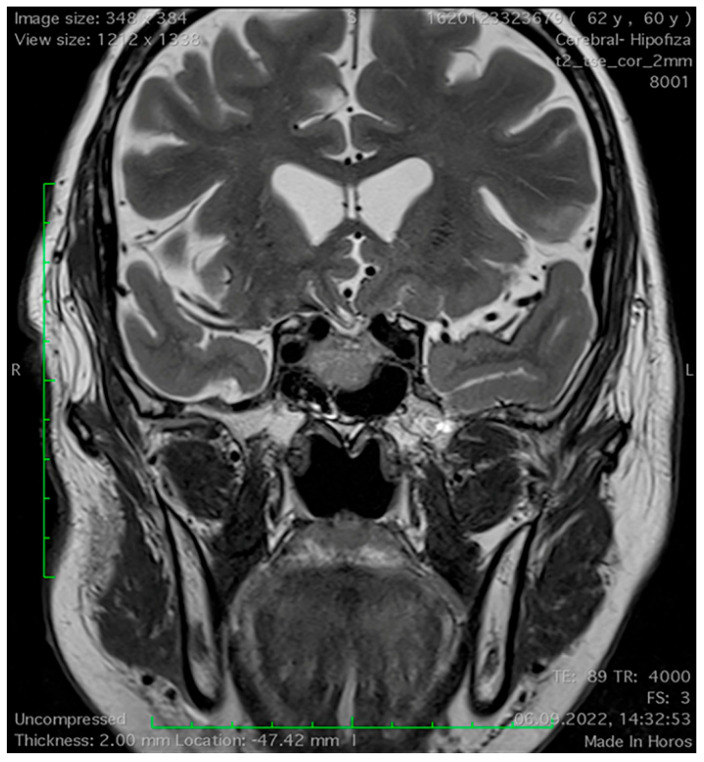
Mucosal thickening of the sphenoid sinus on a coronal T2−weighted magnetic resonance imaging section in a patient with pituitary adenoma.

The MRI features of the sphenoid sinus are depicted in Table 3. As one can observe, the sellar type of SS pneumatisation is the most frequently encountered, and no significant differences in the pneumatisation pattern were detected between both study groups. Mucosal thickness of ≥3 mm of the SS was observed in a greater proportion of the NFPA patients (42.5%) than in the control group (3%), and this difference is statistically significant (Figure 1 and Figure 2). No statistically significant differences between the presence of retention cysts and lateral recesses were detected. The proportion of sphenoethmoidal air cells (Onodi cells) was not different between the two study groups.

**Figure 3:**
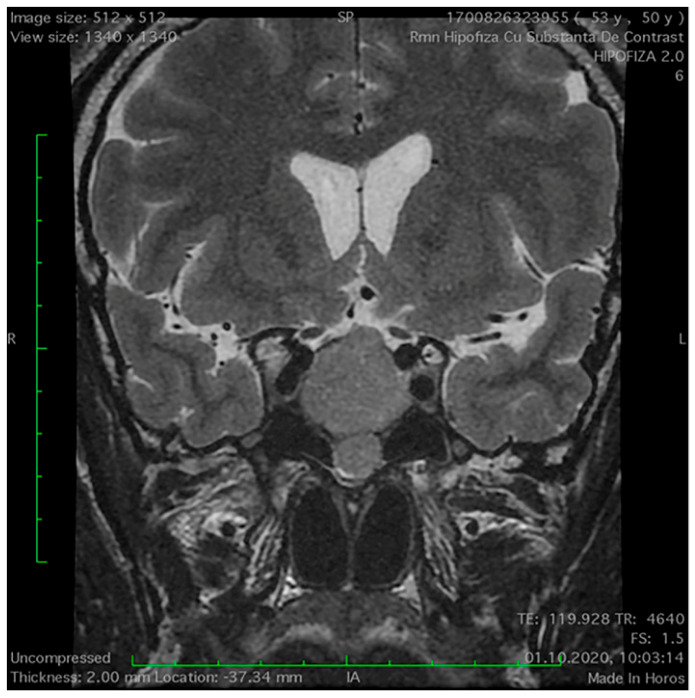
Measurement of the distance between the two optic nerves on a coronal MRI section.

The distances between different anatomic structures are displayed in Table 4. The space between the two optic nerves is statistically significantly greater in the NFPA group as compared to the control group (Figure 3). No significant differences were noted between the intercarotid distances and the distance between the two foramen rotundum in the study groups. The thickness of the adenoid tissue was similar in both study groups.

## 4. Discussion

The sphenoid sinus (SS) holds significant importance as it serves as the primary passage to the pituitary fossa during transnasal transsphenoidal surgery. Positioned centrally within the skull base, the SS exhibits variability in size and shape. It is susceptible to a range of sinus and non-sinus related pathologies, which can manifest through mucosal thickening, fluid accumulation, and partial or complete opacification on radiological imaging. Although isolated pathologies affecting the SS are uncommon, they may occur alongside rhinosinusitis cases, with isolated sphenoiditis reported at a rate of 1% to 3% among affected individuals [12]. 

In accordance with previous reports from the literature, the sellar type of SS pneumatisation was the most-encountered feature in our cohort of patients. Hamberger et al. initially outlined that the pneumatization of the sphenoid bone should be separated into conchal, presellar, or sellar patterns. The conchal pattern, characterized by absent or rudimentary pneumatization of the sphenoid bone, is relatively rare [13]. In the presellar pattern, pneumatization extends to the anterior surface of the sella turcica, while extension to the posterior surface is termed sellar pneumatization [13]. According to Hamberger et al., the sellar configuration is the most prevalent pattern, observed in 86% of individuals, while presellar and conchal patterns account for only 11% and 3% of cases, respectively [13]. The most recent report by Sharif et al. on SS pneumatisation in pituitary tumours also nominated the sellar pattern as the most prevalent subtype, accounting for 91.8% of individuals. However, the findings of Sharif et al. contrast with previous studies in which the prevalence of conchal patterns ranged from 2% to 28%, a discrepancy possibly influenced by racial variations [1]. Conchal and presellar patterns are more common among Chinese individuals compared to other ethnicities [14]. Disparities in study designs, such as those involving cadaveric investigations or imaging, may also contribute to these discrepancies [14]. Investigations involving cohorts of fewer than 200 Caucasian patients have consistently identified sellar pneumatization as the predominant pattern surrounding the sella, with prevalence rates ranging from 65.5% to 98% [3,15]. A modified version of the Hamberger classification has been utilized by some surgeons, which considers a presellar configuration (anterior to the sella turcica), a sellar configuration (parallel to the surface of the sella turcica), and a postsellar configuration (pneumatization below the sella turcica). 

The sphenoethmoidal air cell, named the Onodi air cell, represents the most prevalent anatomical variation among posterior ethmoidal cells [16]. Due to its significant implications during surgeries involving the SS and related surgical procedures such as transsphenoidal hypophysectomy and parasellar surgery, the presence of the sphenoethmoidal air cell holds paramount importance. The prevalence of the Onodi air cell exhibits considerable variability in both clinical anatomical and radiological studies (ranging from 42% to 60% clinically and from 8% to 44% radiologically, respectively) [17]. In the presence of the sphenoethmoidal cell, the optic nerve traverses immediately lateral to this ethmoid sinus rather than through the sphenoid sinus itself. A risk for the optic nerve should be mentioned in the presence of this anatomical variant when sphenoidotomy is conducted in a careless manner. The presence of the Onodi air cell can displace the sphenoid sinus downward, thereby reducing its pneumatization and potentially contributing to recurrent sphenoiditis [5]. In our cohort, the Onodi cell was present in 30% of controls and 33% of NFPA patients, similar to data reported in the literature. In a recent report [5], the incidence of Onodi cells in NFPA patients was notably lower compared to the control population. The authors did not propose any hypotheses to explain why the Onodi air cell is less prevalent in patients with NFPA, but its reduced occurrence may potentially be advantageous during transsphenoidal hypophysis surgery [5]. Further randomized controlled studies could offer insights into this controversy.

Although MRI’s utility in evaluating sinonasal disease is limited, radiologists interpreting brain MRIs are typically familiar with the MRI images of sinus inflammation. Reported prevalences of abnormal mucosal thickening on brain MRI studies have varied widely, ranging from 29.5% to 66% [18,19,20,21,22]. Furthermore, inconsistencies exist in the relationship between these findings and factors such as season, age, and sex in previously reported studies.

These discrepancies may stem from climatic differences among the geographic regions where the studies were conducted, impacting detection rates. For instance, Nazri et al. found a 29.5% prevalence of incidental paranasal sinus abnormalities (IPSA) in a Malaysian population, while Hansen et al. reported a 66% prevalence in a Norwegian climate [6,23]. A study conducted in Ankara, Turkey, reported 45.5% abnormal mucosa in the study population. Notably, abnormal MRI sinus scans were significantly higher in winter (50.6%) compared to summer (40.3%) [24]. In our population, we could not detect differences between the two seasons in connection to mucosal thickening.

Previous studies have shown inconsistency regarding the relationship between IPSA frequency and sex. While the majority of these studies found no significant difference between men and women in IPSA frequency, Hansen et al. reported a higher prevalence among men [6]. Consistent with Hansen et al.’s findings, our study also found a significantly higher prevalence of mucosal thickness in maxillary and anterior ethmoid sinuses among men compared to women. Nevertheless, there are studies reporting a male predominance in both acute and chronic sinusitis [25,26]. Ference et al., in their comprehensive review on gender differences in the frequency, management, and quality of life of patients with chronic rhinosinusitis, suggested that women may be more inclined to report symptoms, seek medical assistance, and have a poorer self-assessment of health, potentially leading to an erroneously high prevalence of chronic rhinosinusitis among women [27]. 

Remarkably, we could demonstrate in our population that significant SS mucosa thickening is independent of season and not related to gender in NFPA patients. Moreover, while abnormal MRI maxillary, ethmoid, and frontal scans are equally distributed among study and control groups, SS mucosal thickening is significantly higher only in NFPA patients.

Inconsistencies in the incidence of SS mucosal thickening on preoperative MRI scans among patients diagnosed with pituitary apoplexy and Rathke’s cleft cyst have been reported [7,8,9,10,11]. Only Şahin B et al. explored the association between the presence of NFPA and SS mucosal thickening [5]. These authors reported that over two-thirds of NFPA patients exhibited SS mucosal thickening, in contrast to only 12.5% in the control group. Arita et al. initially documented the connection between SS mucosal thickening and pituitary gland pathology [7]. They analysed MRI results from 14 patients treated for pituitary apoplexy (PA), with acute stage scans available for 11 individuals, 9 of whom (81.8%) showed SS mucosal thickening. In their investigation, the incidence of SS mucosal thickening in control patients, consisting of 58 functioning and 42 non-functioning adenomas, was 15% [7].

Liu et al. conducted a retrospective analysis of 28 patients diagnosed with PA, revealing SS mucosal thickening in 22 individuals (79%). Additionally, they observed a correlation between the presence of mucosal thickening in the SS and the severity of PA [8]. Other authors observed a temporal relationship between MRI-detected SS mucosal thickening and the occurrence of PA. They noted that mucosal thickening might precede the apoplectic event, potentially serving as an early indicator of PA [28]. Waqar et al. investigated the imaging features of 47 PA patients and 50 NFPA patients using MRI scans and found that the incidence of mucosal thickening exceeding 1 mm was 61% in the PA group and 6% in the NFPA group [9]. However, mucosal thickening of 1 mm or less was present in 94% of NFPA patients. Several investigations proposed a possible association between NFPA and mucosal thickening in the SS.

Takasuna et al. conducted a retrospective analysis of 84 patients diagnosed with various pituitary pathologies, comprising 51 with PA, 18 with Rathke’s cleft cyst, and 15 with other tumours. They found that the occurrence of SS mucosal thickening was 16.7% for Rathke’s cleft cyst and 2% for PAs, suggesting that mucosal thickening in the SS is not exclusive to PA but can also manifest in other hypophyseal pathologies such as Rathke’s cleft cyst [11]. Şahin B et al. also demonstrated the presence of SS mucosal thickening in 67.7% (44 out of 65 patients) of individuals with NFPA, which was the first report establishing a connection between SS mucosal thickening and NFPA [5].

Mucosal thickening typically appears as a radiological sign of sphenoid sinusitis having infectious or inflammatory origins [29]. However, SS mucosal thickening can also manifest in certain hypophyseal pathologies like granulomatous hypophysitis and Rathke’s cleft cyst, even in the absence of clinical rhinosinusitis manifestations [11]. One hypothesis concerning the development of SS mucosal thickening suggests that the obstruction of transsellar venous flow leads to vascular congestion and consequently to a sudden increase in intrasellar pressure. This elevated pressure causes the congestion of dural blood flow due to increased cavernous sinus pressure, ultimately leading to mucosal thickening in the SS [7].

Another hypothesis suggests that an infectious process, possibly associated with PA or other pituitary pathologies, could be responsible for SS mucosal thickening. The connection between cavernous sinus thrombosis and sphenoid sinusitis might support this idea. Additionally, it is plausible that this condition could have a neurological or endocrinological origin. However, the precise mechanism underlying mucosal thickening remains unknown. SS mucosal thickening can create challenges during preoperative MRI assessments of patients with NFPAs. This radiological observation might be mistaken for chronic rhinosinusitis, potentially leading neurosurgeons to postpone surgery due to concerns about bacterial contamination [5].

Conversely, in certain instances, communication with the subarachnoid space and cerebrospinal fluid leakage may arise following hypophysis surgery. Hence, it becomes imperative to differentiate whether SS mucosal thickening stems from chronic rhinosinusitis or pituitary adenoma. The absence of rhinosinusitis symptoms like purulent nasal discharge, facial pain or pressure, reduced sense of smell (hyposmia or anosmia), and nasal congestion, alongside the lack of radiological signs of rhinosinusitis, may suggest that mucosal thickening is linked to a pituitary adenoma [5]. 

Another important finding of our study is the statistically significant increased distance between the two optic nerves in the NFPA group. The optic nerves and optic chiasm represent crucial anatomical landmarks during sellar and parasellar surgeries. A recent study assessed the distance between optic nerves in NFPA patients and revealed that the gap between optic nerves is expanded in NFPA patients compared to the controls [5]. The emerged hypothesis considers that compression exerted by the adenoma might impede venous return from the pituitary gland, potentially leading to tissue oedema, swelling, and consequently an enlarged stalk, which could laterally displace the optic nerves, resulting in an increased distance between them [5].

Moreover, a robust positive correlation was observed between adenoma volume and the distance separating the two optic nerves [5], which suggests that the adenoma could elevate the distance between the optic nerves through a direct mass effect [5], but further researchers should validate these hypotheses. 

The ICD (cavernous intercarotid distance) plays a crucial role in transsphenoidal hypophysis surgery as it provides a surgical pathway to the sellar and parasellar regions. The ICD has been investigated using different measurement methods [30,31,32,33,34]. However, only a limited number of studies have specifically examined differences in the ICD between patients with sellar and parasellar pathologies and healthy individuals. Nunes et al. observed that the mean ICD was larger in patients with pituitary giant adenoma compared to both those with macroadenoma and controls. A narrower mean ICD was found for acromegalic patients as compared with healthy individuals [35].

Individuals with pituitary adenomas tend to have a larger ICD compared to healthy persons, with a positive association observed between the diameter of the lesion and the ICD [33]. Data inconsistent with the abovementioned results revealed slightly greater mean ICD-P and ICD-C in patients with NFPA compared to controls, although this disparity did not reach statistical significance [5], possible due to the broad spectrum of adenoma sizes and the prevalence of microadenomas among the patients [5]. 

However, the important limitations of the present study deserve a thorough discussion: retrospective study, small sample size, lack of follow-up, heterogenous dimensions of pituitary tumours.

Because of the retrospective nature of our study, we could not track the presence of respiratory allergy, smoking status, inferior respiratory diseases, or blood eosinophilia. All of these factors are well known to account for an increased incidence of mucosal thickening on MRI scans. On the follow-up of these patients, one should document the post-operative status of the thickened SS mucosa and the occurrence of SS infections. Specifically, a recent paper demonstrated that patients undergoing transsphenoidal endoscopic surgery for NFPA were more susceptible to postoperative sinusitis compared to those operated from a functioning adenoma [36]. NFPAs typically remain asymptomatic initially and are often detected in later stages when exerting pressure on surrounding structures [37]. This growth process may lead to heightened chronic inflammation or structural alterations within the sinus cavity even prior to surgical intervention. These pre-existing conditions could potentially predispose individuals to an elevated risk of postoperative sinusitis [36].

Regarding the relationship between inter-optic and intercarotid distances and the mass effect of the tumour, in order to validate this hypothesis, the inclusion of only macroadenomas and the comparison between NFPA and secreting adenomas would be advisable.

The aetiology of sellar SS mucosal thickening in the context of pituitary adenomas remains uncertain. SS thickening typically indicates sphenoid sinusitis, which can have either inflammatory or infectious origins [29]. Currently, there is insufficient evidence to conclusively support either hypothesis. Agrawal et al. presented two cases of patients diagnosed with pituitary adenomas who underwent biopsies of the sphenoid sinus mucosa and reported a moderate degree of inflammation [28]. Comparing these findings to two patients who underwent elective resection of pituitary adenomas without radiological evidence of thickened SS mucosa, they similarly detected a moderate grade of inflammation in the SS mucosa [28]. While drawing definitive conclusions from this limited analysis is challenging, it does suggest that SS mucosal thickening may not solely indicate inflammation. Alternatively, it could be hypothesised that SS mucosal thickening reflects an infective process potentially linked to the development of NFPA. Supporting this idea, a case of cavernous sinus thrombosis secondary to sphenoid sinusitis has been documented [38]. Presently, employing the MALDI-TOF mass spectrometry method, we are investigating the microbiological flora of patients with NFPA compared to those with other pituitary tumours [39]. It is plausible that erosion of the sellar dura and bony floor, frequently observed in NFPA cases, may facilitate the spread of inflammation or infection. Additionally, histology and detection of aquaporins are currently performed on SS mucosa sampled during surgery. 

## 5. Conclusions

In the present study we noticed that the sellar type was the predominant type of pneumatization of the SS encountered. Additionally, we have demonstrated a statistically significant association between the presence of pituitary tumour and mucosal thickening of the SS mucosa, independent of the patient gender and season of examination. The distance between the two optic nerves is statistically higher in NFPA patients. One can speculate that in patients without signs of rhinosinusitis, SS mucosal thickening on an MRI scan should prompt the investigation of a possible pituitary adenoma. The findings of this study could be useful in order not to delay a neurosurgical approach in patients with the thickening of SS mucosa without signs of rhinosinusitis.

## Figures and Tables

**Table 1:** Mucosal thickening ≥ 1 mm in each sinus.

Group	M (%)	AE (%)	PE (%)	F (%)	S (%)
Adenoma	12 (30%)	11 (27%)	9 (22%)	4 (10%)	26 (65%)
Control	8 (26%)	7 (23%)	5 (16%)	4 (13%)	7 (23%)
*p* value	0.75	0.63	0.51	0.68	0.0005

Abbreviations: M, maxillary sinus; AE, anterior ethmoid sinus; PE, posterior ethmoid sinus; F, frontal sinus; S, sphenoid sinus.

**Table 2:** Relationship of mucosal thickening of ≥1 mm with gender and season.

Variable	M	AE	PE	F	S
Male	14	12	8	5	14
Female	6	6	6	3	19
*p* value	0.001	0.002	0.09	0.12	0.15
Summer	9	7	6	3	16
Winter	11	11	8	5	17
*p* value	0.38	0.17	0.25	0.41	0.32

Abbreviations: M, maxillary sinus; AE, anterior ethmoid sinus; PE, posterior ethmoid sinus; F, frontal sinus; S, sphenoid sinus.

**Table 3:** Sphenoid sinus characteristics in both study groups.

Parameter	Adenoma Group	Control Group	*p* Value
Pneumatization type			*p* = 0.859
Sellar	34	24	
Presellar	5	5	
Conchal	1	1	
Mucosal thickness			*p* = 0.0004
≤1 mm	14	23	
1–3 mm	9	6	
≥3 mm	17	1	
Retention cyst			*p* = 0.457
Present	3	1	
Absent	37	29	
Lateral recess			*p* = 0.516
Present	13	12	
Absent	27	18	
Sphenoethmoidal air cell			*p* = 0.556
Present	13	9	
Absent	27	21	

**Table 4:** The distances between various anatomic structures in both study groups.

Distances (mm) *	Adenoma Group 40 Cases	Control Group 30 Cases	*p* Value
Cavernous intercarotid distance	20.4 ± 3.2	19.1 ± 3.9	*p* = 0.273
Petrous intercarotid distance	16.7 ± 2.8	15.4 ± 3.1	*p* = 0.140
Distance between two ON	17.3 ± 0.81	11.2 ± 0.92	*p* < 0.001
Distance between two FR	32.1 ± 6.1	31.3 ± 5.8	*p* = 0.421
Adenoid tissue thickness	6.1± 3.2	5.7 ± 3.5	*p* = 0.372

Abbreviations: * mean ± standard deviation; ON, optic nerves; FR, foramen rotundum.

## Data Availability

The datasets generated during the current study are available from the corresponding author on reasonable request.

## References

[1] Sharifi G., Ohadi M.A.D., Abedi M., Khajavi M., Shahjouei S., Moradi A., Bahranian A., Dilmaghani N.A. (2023). Surgical Anatomic Findings of Sphenoid Sinus in 1009 Iranian Patients with Pituitary Adenoma Undergoing Endoscopic Transsphenoidal Surgery. Eur. Arch. Otorhinolaryngol..

[2] Kazkayasi M., Karadeniz Y., Arikan O.K. (2005). Anatomic Variations of the Sphenoid Sinus on Computed Tomography. Rhinology.

[3] Dal Secchi M., Dolci R., Teixeira R., Lazarini P. (2018). An Analysis of Anatomic Variations of the Sphenoid Sinus and Its Relationship to the Internal Carotid Artery. Int. Arch. Otorhinolaryngol..

[4] Ntali G., Wass J.A. (2018). Epidemiology, Clinical Presentation and Diagnosis of Non-Functioning Pituitary Adenomas. Pituitary.

[5] Şahin B., Topaloğlu Ö. (2021). Sphenoid Sinus Mucosal Thickening in Patients with Non-functioning Pituitary Adenoma. Int. J. Clin. Pract..

[6] Hansen A.G., Helvik A.-S., Nordgård S., Bugten V., Stovner L.J., Håberg A.K., Gårseth M., Eggesbø H.B. (2014). Incidental Findings in MRI of the Paranasal Sinuses in Adults: A Population-Based Study (HUNT MRI). BMC Ear Nose Throat Disord..

[7] Arita K., Kurisu K., Tominaga A., Sugiyama K., Ikawa F., Yoshioka H., Sumida M., Kanou Y., Yajin K., Ogawa R. (2001). Thickening of Sphenoid Sinus Mucosa during the Acute Stage of Pituitary Apoplexy: Case Report. J. Neurosurg..

[8] Liu J.K., Couldwell W.T. (2006). Pituitary Apoplexy in the Magnetic Resonance Imaging Era: Clinical Significance of Sphenoid Sinus Mucosal Thickening. J. Neurosurg..

[9] Waqar M., McCreary R., Kearney T., Karabatsou K., Gnanalingham K.K. (2017). Sphenoid Sinus Mucosal Thickening in the Acute Phase of Pituitary Apoplexy. Pituitary.

[10] Semple P.L., Jane J.A., Lopes M.B.S., Laws E.R. (2008). Pituitary Apoplexy: Correlation between Magnetic Resonance Imaging and Histopathological Results. J. Neurosurg..

[11] Takasuna H., Sase T., Ito H., Ono H., Oshio K., Tanaka Y. (2017). Clinical Significance of Thickened Sphenoid Sinus Mucosa in Rathke’s Cleft Cyst. Surg. Neurol. Int..

[12] Lawson W., Reino A.J. (1997). Isolated Sphenoid Sinus Disease: An Analysis of 132 Cases. Laryngoscope.

[13] Hamberger C.A., Hammer G., Norlen G., Sjogren B. (1961). Transantrosphenoidal Hypophysectomy. Arch. Otolaryngol. Head. Neck Surg..

[14] Lu Y., Pan J., Qi S., Shi J., Zhang X., Wu K. (2011). Pneumatization of the Sphenoid Sinus in Chinese: The Differences from Caucasian and Its Application in the Extended Transsphenoidal Approach: Pneumatization of the Sphenoid Sinus in Chinese. J. Anat..

[15] Wang J., Bidari S., Inoue K., Yang H., Rhoton A. (2010). Extensions of the Sphenoid Sinus: A New Classification. Neurosurgery.

[16] Chmielewski P.P. (2023). Clinical Anatomy of the Paranasal Sinuses and Its Terminology. Anat. Sci. Int..

[17] Tomovic S., Esmaeili A., Chan N., Shukla P., Choudhry O., Liu J., Eloy J. (2013). High-Resolution Computed Tomography Analysis of Variations of the Sphenoid Sinus. J. Neurol. Surg. B Skull Base.

[18] Tarp B., Fiirgaard B., Christensen T., Jensen J.J., Black F.T. (2000). The Prevalence and Significance of Incidental Paranasal Sinus Abnormalities on MRI. Rhinology.

[19] Rak K.M., Newell J.D., Yakes W.F., Damiano M.A., Luethke J.M. (1991). Paranasal Sinuses on MR Images of the Brain: Significance of Mucosal Thickening. AJR Am. J. Roentgenol..

[20] Jafari-Pozve N., Roshanzamir N. (2018). Association between the Seasonal Changes and Mucous Retention Cyst of Maxillary Antrum in Cone Beam Computed Tomography Images in a Sample Population of Isfahan, Iran. Iran. Indian J. Dent. Res..

[21] Cooke L.D., Hadley D.M. (1991). MRI of the Paranasal Sinuses: Incidental Abnormalities and Their Relationship to Symptoms. J. Laryngol. Otol..

[22] Wani M.K., Ruckenstein M.J., Parikh S. (2001). Magnetic Resonance Imaging of the Paranasal Sinuses: Incidental Abnormalities and Their Relationship to Patient Symptoms. J. Otolaryngol..

[23] Nazri M., Bux S.I., Tengku-Kamalden T.F., Ng K.-H., Sun Z. (2013). Incidental Detection of Sinus Mucosal Abnormalities on CT and MRI Imaging of the Head. Quant. Imaging Med. Surg..

[24] Özdemir M., Kavak R.P. (2019). Season, Age and Sex-Related Differences in Incidental Magnetic Resonance Imaging Findings of Paranasal Sinuses in Adults. Turk. Arch. Otorhinolaryngol..

[25] Lebovics R.S., Moisa I.I., Ruben R.J. (1989). Sex Predilection in Patients with Acute Frontal Sinusitis. Ear Nose Throat J..

[26] Shi J.B., Fu Q.L., Zhang H., Cheng L., Wang Y.J., Zhu D.D. (2015). Epidemiology of Chronic Rhinosinusitis: Results from a Cross-Sectional Survey in Seven Chinese Cities. Allergy.

[27] Ference E.H., Tan B.K., Hulse K.E., Chandra R.K., Smith S.B., Kern R.C. (2015). Commentary on Gender Differences in Prevalence, Treatment, and Quality of Life of Patients with Chronic Rhinosinusitis. Allergy Rhinol..

[28] Agrawal B., Dziurzynski K., Salamat M.S., Baskaya M. (2011). The Temporal Association of Sphenoid Sinus Mucosal Thickening on MR Imaging with Pituitary Apoplexy. Turk. Neurosurg..

[29] Chong V.F.H., Fan Y.F. (1998). Comparison of CT and MRI Features in Sinusitis. Eur. J. Radiol..

[30] Jho H.D., Ha H.G. (2004). Endoscopic Endonasal Skull Base Surgery: Part 1-the Midline Anterior Fossa Skull Base. Minim. Invasive Neurosurg..

[31] Hewaidi G.H., Omami G.M. (2008). Anatomic Variation of Sphenoid Sinus and Related Structures in Libyan Population: CT Scan Study. Libyan J. Med..

[32] Yilmazlar S., Kocaeli H., Eyigor O., Hakyemez B., Korfali E. (2008). Clinical Importance of the Basal Cavernous Sinuses and Cavernous Carotid Arteries Relative to the Pituitary Gland and Macroadenomas: Quantitative Analysis of the Complete Anatomy. Surg. Neurol..

[33] Zada G., Agarwalla P.K., Mukundan S., Dunn I., Golby A.J., Laws E.R. (2011). The Neurosurgical Anatomy of the Sphenoid Sinus and Sellar Floor in Endoscopic Transsphenoidal Surgery: Clinical Article. J. Neurosurg..

[34] Nunes C.F., Cabral G.A.P.S., de Mello J.O., Lapenta M.A., Landeiro J.A. (2016). Pituitary Macroadenoma: Analysis of Intercarotid Artery Distance Compared to Controls. Arq. Neuropsiquiatr..

[35] Ebner F.H., Kuerschner V., Dietz K., Bueltmann E., Naegele T., Honegger J. (2009). Reduced Intercarotid Artery Distance in Acromegaly: Pathophysiologic Considerations and Implications for Transsphenoidal Surgery. Surg. Neurol..

[36] Chang Y.C., Tsao Y.N., Chuang C.C., Li C.Y., Lee T.J., Fu C.H., Wei K.C., Huang C.C. (2024). Risk Factors for Isolated Sphenoid Sinusitis after Endoscopic Endonasal Transsphenoidal Pituitary Surgery. Diagnostics.

[37] Fleseriu M., Karavitaki N. (2018). Non-functioning pituitary adenomas, not all the same and certainly not boring!. Pituitary.

[38] Komatsu H., Matsumoto F., Kasai M., Kurano K., Sasaki D., Ikeda K. (2013). Cavernous Sinus Thrombosis Caused by Contralateral Sphenoid Sinusitis: A Case Report. Head Face Med..

[39] Jeican I.I., Barbu Tudoran L., Florea A., Flonta M., Trombitas V., Apostol A., Dumitru M., Aluaș M., Junie L.M., Albu S. (2020). Chronic Rhinosinusitis: MALDI-TOF Mass Spectrometry Microbiological Diagnosis and Electron Microscopy Analysis; Experience of the 2nd Otorhinolaryngology Clinic of Cluj-Napoca, Romania. J. Clin. Med..

